# Where did the super-small sized large bowel advanced cancer come from?

**DOI:** 10.1186/1477-3163-6-7

**Published:** 2007-04-30

**Authors:** Ryo Wada, Koichi Sato, Takafumi Ichida, Hiroshi Maekawa, Kaoru Ogawa, Takeo Maekawa

**Affiliations:** 1 Department of Pathology, Juntendo Shizuoka Hospital of Juntendo University School of Medicine, Shizuoka, Japan; 2 Department of Surgery, Juntendo Shizuoka Hospital of Juntendo University School of Medicine, Shizuoka, Japan; 3 Department of Gastroenterology, Juntendo Shizuoka Hospital of Juntendo University School of Medicine, Shizuoka, Japan

## Abstract

Our study suggested that the super-small sized (less than 15 mm in maximum diameter) large bowel advanced cancers, which were sometimes found, were derived from the superficial depressed-type or flat elevation-type of the colorectal early cancers, not polyp-type of those.

## Background

Although most of the large bowel advanced cancers (the carcinoma invaded into the muscularis propria or deeper zone by Japanese criteria [1]), or T2 and T3 by UICC classification [2]) are 3 – 6 cm in maximum (max.) diameter, those less than 2 cm in max. diameter were sometimes found, today. However, nobody knows what is the pre-lesion of these small sized large bowel advanced cancer.

In the current study, using the characteristics of the histological features of the large bowel cancers with invasion to the submucosal layer (SM-C), the histogenesis of the super-small sized large bowel advanced cancers less than 15 mm in max. diameter (SS-AC) were analyzed pathologically, and some findings were revealed for clearing the question described above.

## Methods

**Figure 1:**
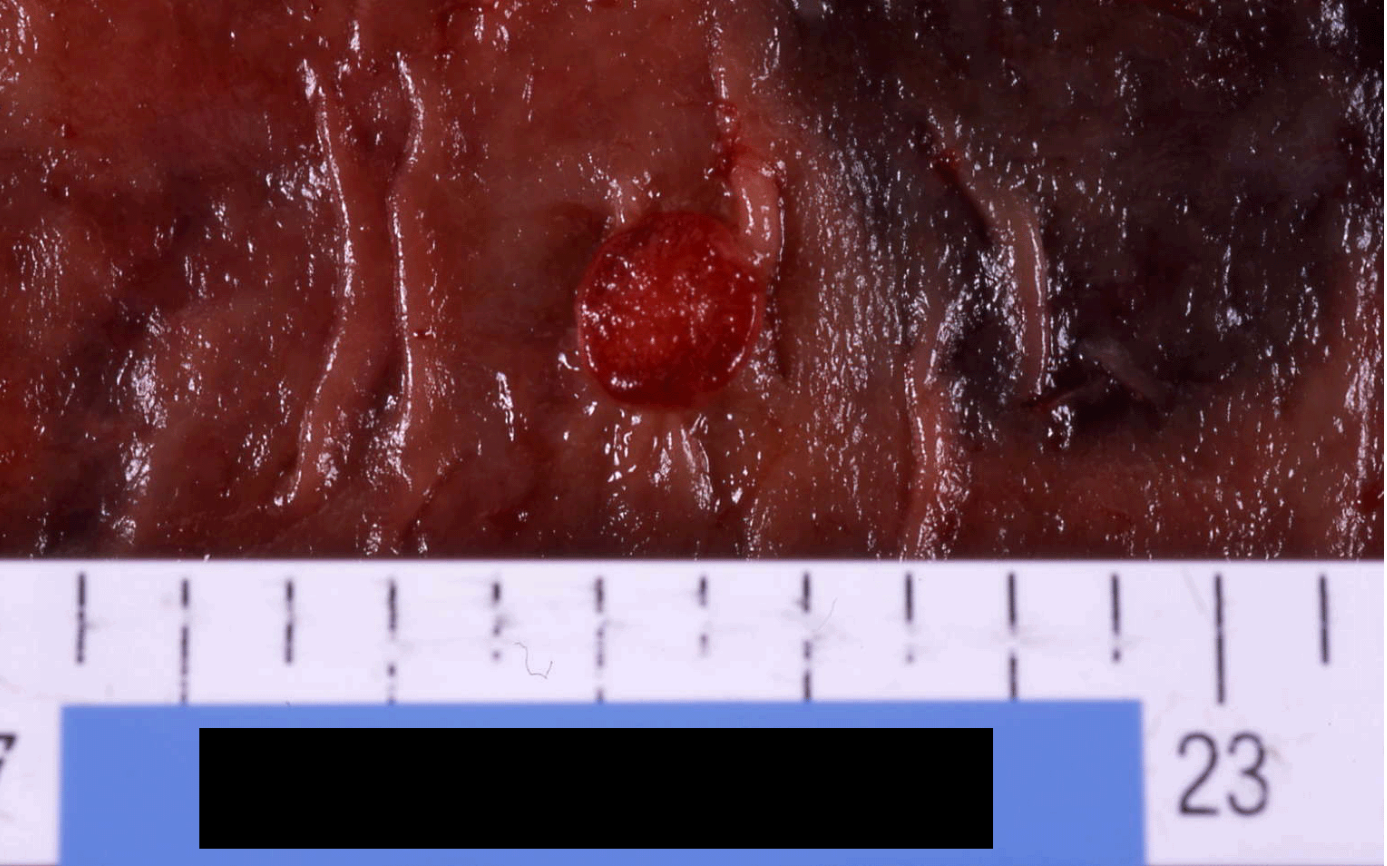
Typical macroscopic finding of the super-small sized large bowel advanced cancers less than 15 mm in max. diameter.

**Figure 2:**
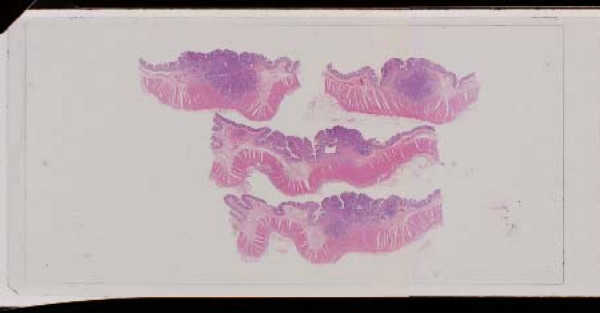
Typical microscopic finding of the super-small sized large bowel advanced cancers less than 15 mm in max. diameter (HE).

Eight cases of the SS-AC lesions (Figures 1 &2) and 65 cases of the SM-C lesions, assessed at the Department of Pathology, Juntendo University Shizuoka Hospital, between 2003 and 2005, were the objects in the current study. In all cases, the informed consent for medical examinations were obtainted from each patient.

The colorectal specimens described above were fixed in 10% buffered formalin solution and prepared by cutting from the entirety of neoplastic area into 1 – 3 mm wide sections. Each section was embedded in paraffin, and stained with hematoxylin and eosin (HE) and anti-a-smooth muscle actin antibody staining (monoclonal mouse antibody, anti-human smooth muscle actin, DAKO, at a dilution of 1:50) by immunohistochemical examination, which were performed by the avidin-biotin-peroxidase-complex method, for light microscopic observation.

**Figure 3:**
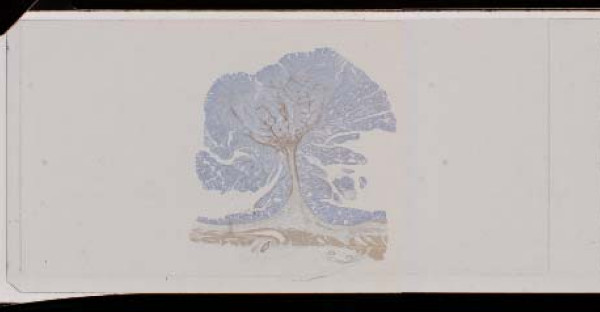
The elevation of muscularis mucosae was seen in some cases of the large bowel cancers with invasion to the submucosal layer (anti-a-smooth muscle actin antibody staining).

SM-C were subclassified 33 cases of SM-C-mm (+) and 32 cases of SM-C-mm(-), if the elevation of muscularis mucosae, which was usually seen in the polyp-type colorectal cancer, were detected in SM-C lesions, they were subclassified as SM-C-mm(+) (Figure 3), and if it were not detected, they were suclassified as SM-C-mm(-).

## Results

### 1. SS-AC

SS-AC was 3.3% of the large bowel advanced cancers in our hospital at same periods (8/245 cases). Among 8 cases of SS-AC, 6 cases were T2 and 2 cases were T3 by UICC classification. Many of SS-AC existed in the left side of large bowel (Transverse colon: 1 case, Descending colon: 1 case, Sigmoid colon: 3 cases, Rectum: 3 cases). The mean size of SS-AC was 12.6 mm (from 10 to 15 mm). Macroscopically, 7 cases were the ulcerative type like Borrmann 2 and one case was the elevation type like Borrmann 1. Microscopically, the carcinoma lesions in all cases showed adenocarcinoma with no adenoma element, and revealed predominantly moderately differentiated pattern in 7 cases, predominatly well differentiated pattern in a case. In all SS-AC, no metastasis of carcinoma to the lymph node were found, although the carcinomatous invasion to the lympho-vessel were detected (lympho-vessel invasion (+): 8 cases, blood-vessel invasion (+): 7 cases). The carcinoma elements within the mucosa in all SS-AC lesions showed the depressed pattern like superficial depressed-type early cancer, and the elevation of muscularis mucosae was not detected in all SS-AC cases.

### 2. SM-C

Most of the SM-C-mm(+) lesions were the elevated lesions (Is, Ip, Ips by Japanese endoscopical classification (1)) and the adenoma elements were found histologically in 76% of them. Seventy % of the SM-C-mm(-) lesions were superficial lesions (IIa or IIc by Japanese endoscopic classification) and no adenoma elements were detected in 72% of them. The mean of the max. size of the SM-C-mm(-) lesions (13.4 ± 5.2 mm) were smaller than that of the SM-C-mm(+) lesions (18.7 ± 8.4 mm) (p > 0.01, t-test).

## Discussion

Our results in the current study showed that the super-small sized large bowel advanced cancers less than 15 mm in max. diameter (SS-AC), which had the abilities of the distant metastasis as high frequencies of the invasion to the vessels, resembled the SM-C-mm(-) lesions and differed from the SM-C-mm(+) lesions, macroscopically and microscopically.

Two hypotheses about the histogenesis of the large bowel cancer have been proposed for many years. One hypothesis, called the polyp-cancer sequence (adenoma-carcinoma sequence) [3], postulates that an adenocarcinoma develops through or on the basis of an adenoma, is sweeping the world. Generally, the polyp-cancer have the elevation of muscularis mucosae like the SM-C-mm(+) lesions in the current study. Another hypothesis claims that an adenocarcinoma arises directly from non-neoplastic colorectal mucosal glands, is generally called the de novo theory [4]. The carcinomas, which developed in the de novo theory, has been pointed out to invade to the submucosal layer even if the size of those lesions were within 5 mm, and showed superficial depressed type or flat elevation type, macroscopically [5-8].

Therefore, there may be little ability that the polyp-cancer is pre-lesion of the SS-AC lesions. On the other hand, there are little inconsistency that the superficial depressed type or the flat elevated type of the early colorectal cancer is the pre-lesion of the SS-AC lesions.

Although the prospective studies may bring out true mechanism of the development of the large bowel cancer, these methods should be avoided at the moral. The current study has brought out the speculation that the pre-lesions of the small sized large bowel advanced cancers are the early cancers with no the elevation of muscularis mucosae, which almostly equal to the superficial depressed or flat elevation types of the early colorectal cancers.
